# Stearoyl-CoA desaturase 1 (SCD1) facilitates the growth and anti-ferroptosis of gastric cancer cells and predicts poor prognosis of gastric cancer

**DOI:** 10.18632/aging.103598

**Published:** 2020-07-29

**Authors:** Chao Wang, Min Shi, Jun Ji, Qu Cai, Qianfu Zhao, Jinling Jiang, Jing liu, Huan Zhang, Zhenggang Zhu, Jun Zhang

**Affiliations:** 1 Department of Oncology, Ruijin Hospital, Shanghai Jiao Tong University School of Medicine, Shanghai 200025, China; 2 Shanghai Institute of Digestive Surgery, Ruijin Hospital, Shanghai Jiao Tong University School of Medicine, Shanghai 200025, China; 3 Department of Radiology, Ruijin Hospital, Shanghai Jiao Tong University School of Medicine, Shanghai 200025, China

**Keywords:** SCD1, gastric cancer, ferroptosis, lipid metabolism, proliferation

## Abstract

Cancer cells are characterized by metabolic alterations. Thereinto, Stearoyl-CoA Desaturase 1 (SCD1), an enzymatic node located in the conversion of saturated fatty acids into monounsaturated fatty acids (MUFAs), has been reported to accelerate the tumorigenesis of multiple cancers. However, its role in the metabolic process of gastric cancer remains largely unexplored. In this study, by *in vitro*, *in vivo* and *in silico* assessments, our results revealed that SCD1 exhibited the ability to promote tumor growth, migration and anti-ferroptosis of gastric cancer. The underlying mechanism might involve the alteration of cancer stemness and modulation of cell cycle-related proteins. Moreover, based on our findings, high expression of SCD1 might predict poor prognosis in gastric cancer patients. Our study provided new insights into the potential of SCD1 as a biomarker as well as a therapeutic target in the treatment of gastric cancer.

## INTRODUCTION

A century ago, the Weltanschauung of doctors and scientists in this field expanded by the “Warburg effect” theory, which described a metabolic shift from oxidative state to glycolytic metabolism existed in cancer cells [1]. Nowadays, the metabolic community have never viewed the “Warburg effect” theory which accompanied with nucleotide, lipids, and protein metabolism solely as “energy generation”. Meanwhile, the heterogeneous and comprehensive interactions in metabolic layer involving in a variety of other cellular processes reminds largely unexplored, especially in cancer cells [1, 2].

Carcinoma is a disorder characterized by increasing metabolic activity which lead to elevate cell proliferation. Therefore, the metabolic characteristic of cancer cells are different from normal cells [3]. One such feature of cancer cell is *de novo* dysregulation of fatty acid biosynthesis, while normal cells dependent on those from exogenous sources. Various enzymes that regulate fatty acid and lipid synthesis were transcriptionally upregulated in tumors [4]. Although clinical trials for lipogenesis inhibitors are ongoing, the regulation and function of lipids in tumors remained elusive.

Human Stearoyl-CoA Desaturase (SCD), is an endoplasmic reticulum associated enzyme in the *de novo* synthesis of fatty acid synthase (FAS) that catalyzes the desaturation of saturated fatty acids (SFAs) to Δ9-monounsaturated counterparts (MUFAs), such as stearic acid (18:0) and palmitic acid (16:0) to oleic acid (18:1) and palmitoleic acid (16:1) [5]. Both SFAs and MUFAs are major components of human cell lipids as basic components of biological membranes and sources of energy and signaling molecules, including cholesteryl esters (CEs) [6]. There are two isoforms of SCD in human tissues and SCD1 is the most abundant ones which expressed in all types of cells. Moreover, SCD1 expression is obviously elevated in many kinds of human cancer and emerged as a novel key player in tumorigenesis [5, 7, 8]. However, seldom studies reported the function of SCD1 in gastric cancer and the underlying mechanisms remains largely unknown.

In this study, the overexpression of SCD1 in tissues of gastric cancer patients was observed, and the function in tumorigenesis were investigated by using manipulation of genetic expression and bio-informational analysis methods.

## RESULTS

### SCD1 expression was frequently dysregulated in a variety of cancers

**Figure 1:**
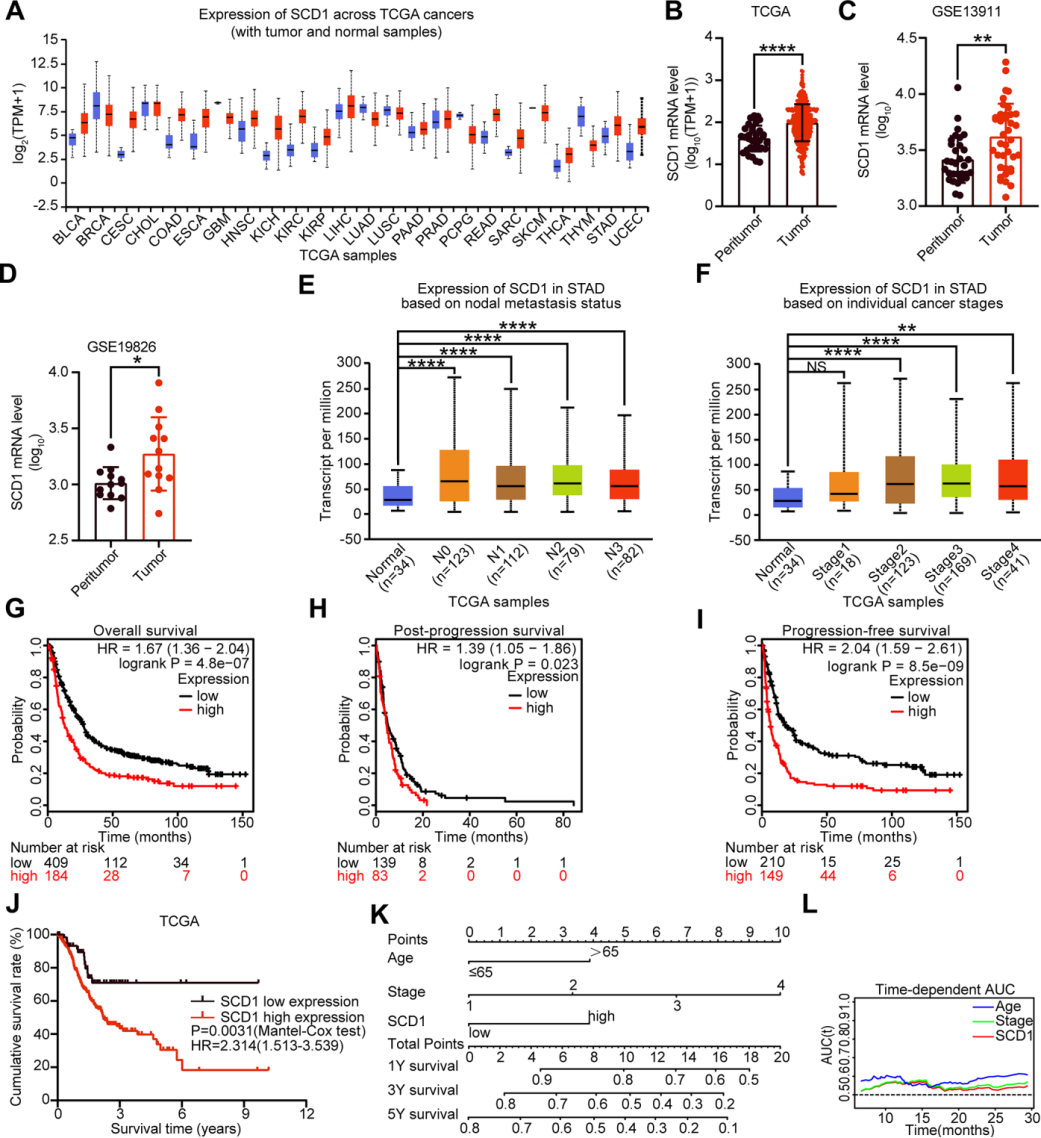
**Expression level and prognostic value of SCD1 in various types of cancer.** (**A**) SCD1 mRNA expression level in pan-cancer tissues and normal tissues. BLCA, bladder urothelial carcinoma; BRCA, breast invasive carcinoma; CESC, cervical squamous cell carcinoma and endocervical adenocarcinoma; CHOL, cholangio carcinoma; COAD, colon adenocarcinoma; ESCA, esophageal carcinoma; GBM, glioblastoma multiforme; HNSC, head and neck squamous cell carcinoma; KICH, kidney chromophobe; KIRC, kidney renal clear cell carcinoma; KIRP, kidney renal papillary cell carcinoma; LIHC, liver hepatocellular carcinoma; LUAD, lung adenocarcinoma; LUSC, lung squamous cell carcinoma; PAAD, pancreatic adenocarcinoma; PRAD, prostate adenocarcinoma; PCPG, pheochromocytoma and paraganglioma; READ, rectum adenocarcinoma; SARC, sarcoma; SKCM, skin cutaneous melanoma; THCA, thyroid carcinoma; THYM, thymoma; STAD, stomach adenocarcinoma; UCEC, uterine corpus endometrial carcinoma; (**B**) SCD1 mRNA expression were significantly overexpressed in gastric cancer tissues compared with peritumor tissues in TCGA database. (**C**, **D**) SCD1 mRNA expression were significantly overexpressed in gastric cancer tissues compared with normal ones in GSE13911 and GSE19826 database. (**E**) The mRNA expression level of SCD1 in different lymph node metastasis based on TCGA-STAD database. (**F**) The mRNA expression level of SCD1 in different AJCC stages based on TCGA-STAD database. (**G**) Overall survival of patients in SCD1-low expression group and SCD1-high expression group based on GEO database. (**H**) Post-progression survival of patients in SCD1-low expression group and SCD1-high expression group based on GEO database. (**I**) Progression-free survival of patients in SCD1-low expression group and SCD1-high expression group based on GEO database. The survival time of patients was compared between groups using the Mantel-Cox test. *, *P* <0.05; **, *P* <0.01; ***, *P* <0.001; ****, *P* <0.0001, respectively. (**J**) Overall survival of patients in SCD1-low expression group and SCD1-high expression group based on TCGA-STAD database. (**K**) Gastric cancer nomogram of overall survival for patients in TCGA-STAD database was examined by adding up of the points identified on the points scale for each characteristics. The total points existed on the bottom scales stand for the probability of 1-, 3- and 5- year survival. (**L**) Time-dependent area under the curve (AUC).

To determine the expression of SCD1 in different cancer types, TCGA database was utilized to identify the mRNA expression level of SCD1. Compared with those in normal tissues, SCD1 mRNA expression was dysregulated in most kinds of cancer tissues, such as in BLCA, CESC, COAD, ESCA, HNSC, KICH, KIRC, KIRP, STAD (*P* <0.0001) and so on. On the contrary, SCD1 expression was suppressed in THYM, PCPG, LUAD, GBM and BRCA (Figure 1A, 1B). To further validate the SCD1 expression in gastric cancer, GEO databases (GSE13911 and GSE19826) were determined to validation. As expected, the SCD1 mRNA expression was higher in gastric cancer tissues than that in normal ones (*P* <0.01 and *P* <0.05; Figure 1C, 1D). Furthermore, SCD1 expression was higher in each status of lymph node metastasis than that in normal tissues (Figure 1E). Additionally, the gastric cancer patient belongs to AJCC stage I had no higher expression of SCD1 than those in normal ones, while AJCC stage II, III and IV groups had relatively higher expression of SCD1 (Figure 1F). The exotic expression of genes might play vital roles in cancers [9], and Kaplan-Meier analysis revealed that SCD1 high-expressed patients have relatively less-optimistic prognostic outcome in terms of overall survival (OS), post-progression survival (PPS) and progression-free survival (PFS) (OS: *P* <0.0001, HR = 1.67 (1.36-2.04); PPS: *P* = 0.023, HR = 1.39 (1.05-1.86); PFS: *P* <0.0001, HR = 2.04 (1.59-2.61); Figure 1G–1I). Meanwhile, the TCGA-STAD dataset also confirmed that SCD1 high-expressed patients had relatively shorter overall survival time (SCD1 high-expressed group: 3.85 ± 0.42 months, SCD1 low-expressed group: 7.10 ± 0.60 months; *P*=0.0031, HR: 2.314, 95% CI: 1.513-3.539; Figure 1J).

**Figure 2:**
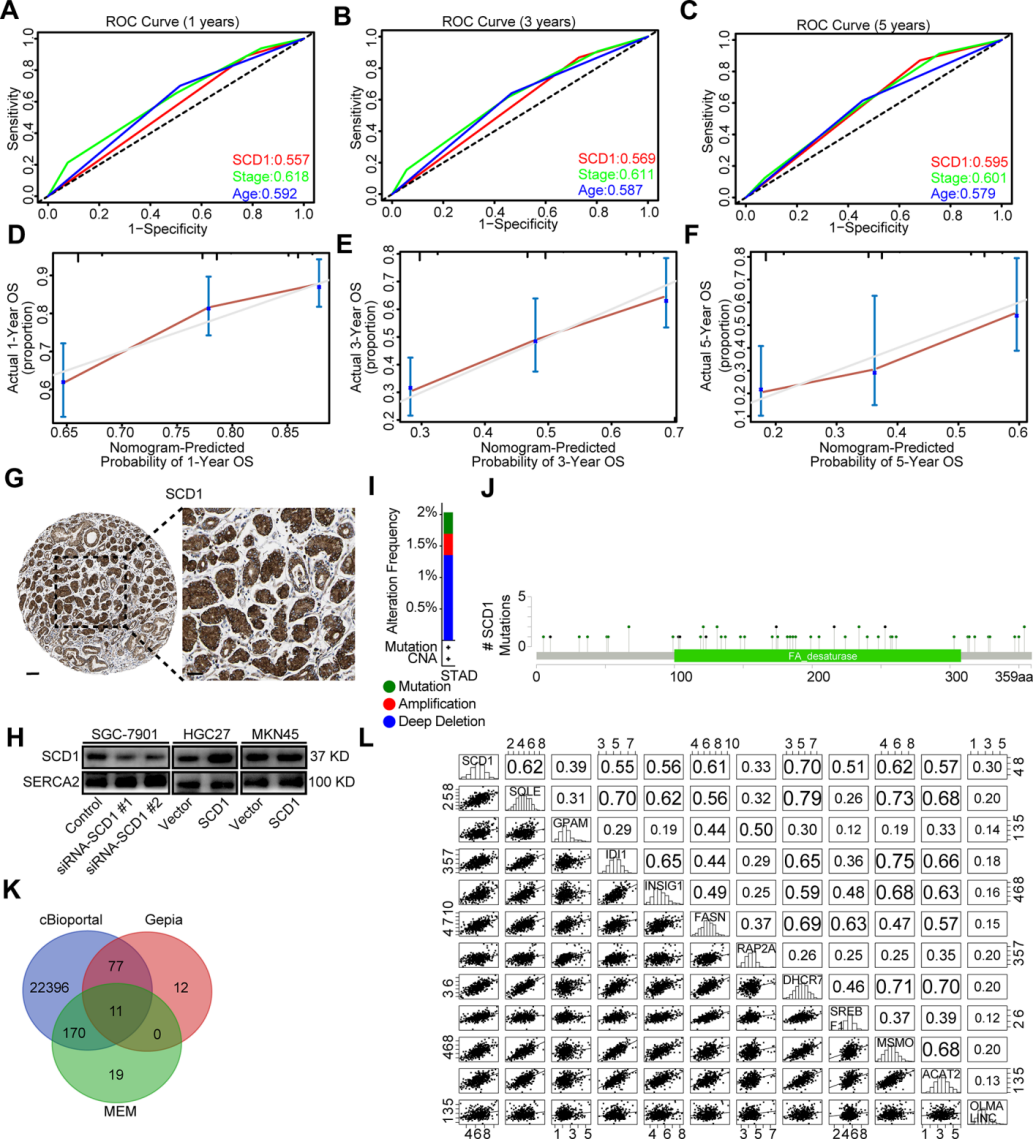
**Bioinformatic analyses of SCD1.** (**A**–**C**) Receiver operating characteristic (ROC) curve at 1, 3 and 5 years according to SCD1 gene expression in the TCGA-STAD database. (**D**–**F**) The calibration curve for predicting overall survival at 1-, 3- and 5-years in the TCGA-STAD database. (**G**) Representative images from gastric cancer tissue stained with SCD1, the scale bar, 100 μm and 50 μm, respectively. (**H**) Endoplasmic reticulum of gastric cancer cells were isolated, proteins were separated by SDS-PAGE and evaluated by immunoblots. (**I**) The alteration frequency of SCD1 were determined by using the cBioportal. (**J**) Screenshot of SCD1 mutation frequencies. (**K**) The Venn diagram illustrated the common correlated factors of SCD1 identified via cBioportal, Gepia and MEM analysis tools. (**L**) The correlations between SCD1 and correlated factors mRNA expression levels in human gastric cancer tissues (TCGA, *n*=375).

To evaluate the prognostic value of SCD1 more comprehensively, the analysis of time-dependent area under the curve (AUC) were performed. The AUC value at 1, 3, and 5 years showed that SCD1 exhibited relatively high C-index values (1-year: 0.557, 3-year: 0.569, and 5-year: 0.595; Figures 1L, 2A–2C). To generate a more accurate predictive model, the SCD1, AJCC Stage and Age were utilized to construct a prognostic nomogram, and the C-index for OS prediction of the formulated nomogram in TCGA-STAD database was 0.649 (95% CI: 0.599-0.709; *P* <0.0001). As shown in Figure 1K, the nomogram predicting 1-, 3- and 5- year overall survival was constructed based on AJCC Stage, Age and SCD1 with hazard ratios. The nomogram calculated the likelihood of survival by adding up the scores identified on the points scale for the three factors. The total score existed on the bottom scale represented the likelihood of 1-, 3- and 5-year survival. The calibration plot for the likelihood of 1-, 3- and 5-year survival showed that optimal agreement between the prediction by nomogram and actual observation (Figure 2D–2F). These results revealed that high expression of SCD1 predicted poor survival in gastric cancer.

### The correlation between SCD1 expression and clinicopathological characteristics

To evaluate the correlation of SCD1 with tumor biology, comparisons of the clinicopathologic features with SCD1 expression were investigated based on TCGA-STAD database. As shown in Table 1, high expression of SCD1 were positively associated with elder age (*P*=0.015) and male (*P*=0.023). It is, thus, of interest to investigate whether SCD1 is an independent prognostic risk factor for gastric cancer. TCGA-STAD database and COX regression hazard model were employed to perform univariate and multivariate analysis, and outcome, which adjust T phase, Age, AJCC stage and other factors, confirmed the negative correlation between higher SCD1 expression and shorter survival (hazard ration (HR): 2.170; 95% confidence interval (CI): 1.247-3.777; *P*<0.006 and HR: 1.821; 95% CI: 1.026-3.232; *P*<0.041; respectively; Table 2).

**Table 1:** Correlations between SCD1 expression and clinical characteristics in TCGA-STAD database.

**Characteristics**	**SCD1 expression (case No.)**		***P* value**
	**Low level**	**High level**	
Gender			
Male	35	183	0.023
Female	33	93	
Age(year)			
≤65	39	113	0.015
>65	29	163	
T phase			
I	3	13	0.767
II	16	54	
III	33	128	
IV	16	81	
Lymph node metastasis			
0	24	83	0.522
I	20	68	
II	12	60	
III	12	65	
AJCC Stage			
I	11	38	0.123
II	29	81	
III	22	126	
IV	6	31	

**Table 2:** Univariate and multivariate analyses of characteristics associated with overall survival in TCGA-STAD database.

**Characteristics**	**Univariable overall survival**				**Multivariable overall survival**			
	**Hazard Ratio**	**95.0% Confidence limits**		***P* value**	**Hazard Ratio**	**95.0% Confidence limits**		***P* value**
		**Lower**	**Upper**			**Lower**	**Upper**	
Gender	0.817	0.568-1.174		0.274	/	/	/	/
T1 (reference)	1							
T2	5.527	0.747-40.890		0.094	4.438	0.572-34.457		0.154
T3	7.574	1.051-54.580		0.045	6.483	0.753-55.789		0.089
T4	7.654	1.050-55.805		0.045	5.561	0.632-48.917		0.122
N0 (reference)	1							
N1	1.468	0.902-2.388		0.122	1.383	0.706-2.708		0.344
N2	1.493	0.883-2.523		0.134	1.362	0.597-3.107		0.463
N3	2.398	1.488-3.865		0.0003	2.008	0.902-4.470		0.088
AJCC Stage 1(reference)	1							
AJCC Stage 2	1.576	0.797-3.118		0.191	0.892	0.334-2.385		0.820
AJCC Stage 3	2.191	1.153-4.164		0.017	0.816	0.223-2.987		0.759
AJCC Stage 4	4.067	1.980-8.354		0.0001	1.862	0.519-6.680		0.340
Age	1.696	1.190-2.416		0.003	1.814	1.249-2.635		0.002
SCD1	2.170	1.247-3.777		0.006	1.821	1.026-3.232		0.041

N, lymph node metastasis; T, T phase.

### Bioinformatics analyses of SCD1 and its relative factors

**Figure 3:**
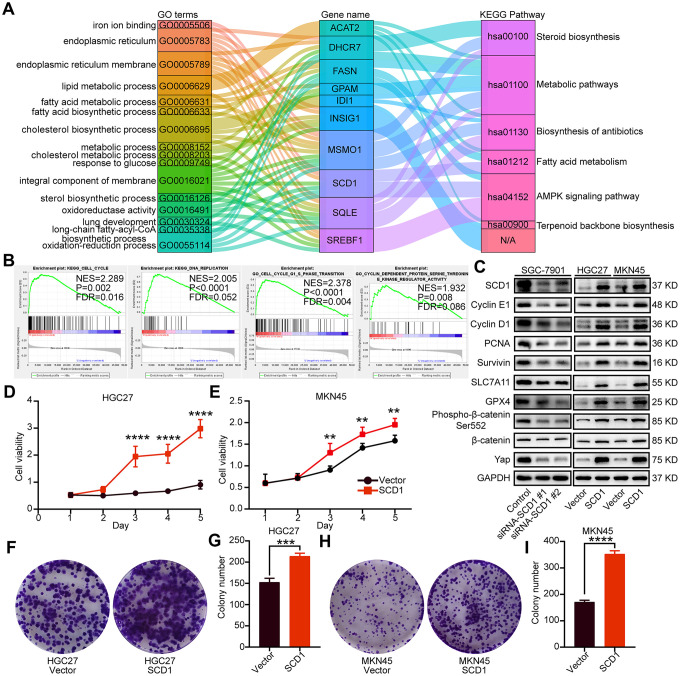
**Oncogenic function of SCD1 in gastric cancer cells.** (**A**) The KEGG pathways and GO terms participated by SCD1 and related factors with *P* value < 0.05. (**B**) The KEGG pathways and GO terms identified via gene set enrichment analysis of tissues with high and low SCD1 expression levels. (**C**) The proteins participated in “DNA replication”, “cell cycle”, “cell cycle G1-S phase transition” and “cyclin dependent protein serine threonine kinase regulator activity”, anti-ferroptosis markers as well as Wnt/β-catenin and Hippo signaling pathways were analyzed using western blotting with the indicated antibodies. GAPDH was used as the internal protein loading control. Each experiment was examined in triplicates. (**D**, **E**) SCD1 promoted proliferation of gastric cancer cells. (**F**–**I**) SCD1 promoted colony formation of gastric cancer cells. Each experiment was examined in triplicate. *, *P* <0.05; **, *P* <0.01; ***, *P* <0.001; ****, *P* <0.0001, respectively.

SCD1 has been reported to locate at endoplasmic reticulum and serve as an integral membrane protein [10]. In order to investigate the protein expression of SCD1 in gastric cancer extensively, the Human Protein Atlas (HPA) database was employed and revealed that SCD1 was highly expressed in the cytoplasm of gastric cancer cells (Figure 2G). Furthermore, SCD1 was ectopically expressed in HGC27 and MKN45 cells and silenced in SGC-7901 cells. Subsequently, the endoplasmic reticulum of these gastric cancer cells were isolated and SCD1 expression was evaluated by immunoblots (Figure 2H). Gene alteration of SCD1 in gastric cancer was analyzed by using cBioportal web tool, and results revealed that deep deletion of SCD1 was the most common type of gene alteration. Meanwhile, minor changes in the amplification and mutation of SCD1 gene were observed (Figure 2I). Noteworthy, SCD1 mutation mainly existed in the fat-acid (FA) desaturase domain of SCD1 (Figure 2J). To better understand the biological function of SCD1, the cBioportal, MEM and Gepia web tools were applied to search for the correlated factors of SCD1. As the Venn diagram shown, there were 11 shared factors among three datasets, including SQLE (Squalene Epoxidase, which is considered to be a rate-limiting enzyme in steroid biosynthesis), GPAM (Glycerol-3-Phosphate Acyltransferase, Mitochondrial, which participates into glycerolipid biosynthesis), IDI1 (Isopentenyl-Diphosphate Delta Isomerase 1, which catalyzes the 1,3-allylic rearrangement of the homoallylic substrate isopentenyl to its highly electrophilic allylic isomer, dimethylallyl diphosphate), INSIG1 (Insulin Induced Gene 1, which regulates cholesterol metabolism, lipogenesis, and glucose homeostasis), FASN (Fatty Acid Synthase, which catalyzes the synthesis of palmitate from acetyl-CoA, malonyl-CoA and NADPH), RAP2A (Small GTP-binding protein which cycles between a GDP-bound inactive and a GTP-bound active form and might regulate cytoskeletal rearrangements, cell migration, cell adhesion and cell spreading), DHCR7 (7-Dehydrocholesterol Reductase, which catalyzes the conversion of 7-dehydrocholesterol to cholesterol), SREBF1 (Sterol Regulatory Element Binding Transcription Factor 1, which regulates transcription of the LDL receptor gene as well as the fatty acid and to a lesser degree the cholesterol synthesis pathway), MSMO1 (Methylsterol Monooxygenase 1, which locates to the endoplasmic reticulum membrane and might play a role in cholesterol biosynthesis), ACAT2 (Acetyl-CoA Acetyltransferase 2, which involves in the biosynthetic pathway of cholesterol), OLMALINC (Oligodendrocyte Maturation-Associated Long Intergenic Non-Coding RNA) (Figure 2K). Then, the relationship between mRNA expression of SCD1 and these 11 correlated factors were examined via using TCGA-STAD database. SCD1 were positively correlated with SQLE (R = 0.62, *P* <0.0001), GPAM (R = 0.39, *P* <0.0001), IDI1 (R = 0.55, *P* <0.0001), INSIG1 (R = 0.56, *P* <0.0001), FASN (R = 0.61, *P* <0.0001), RAP2A (R = 0.33, *P* <0.0001), DHCR7 (R = 0.70, *P* <0.0001), SREBF1 (R = 0.51, *P* <0.0001), MSMO1 (R = 0.62, *P* <0.0001), ACAT2 (R = 0.57, *P* <0.0001), OLMALINC (R = 0.30, *P* <0.0001; Figure 2L). Consistent with the outcome of three web tools, KEGG pathways and functional enrichment clustering of 11 correlated factors and SCD1 showed that several KEGG pathways and GO terms were identified to be of significance (*P* <0.05), such as, iron ion binding, endoplasmic reticulum membrane, fatty acid metabolism, AMPK signaling pathway (Figure 3A).

### SCD1 promoted growth and anti-ferroptosis of gastric cancer cells

**Figure 4:**
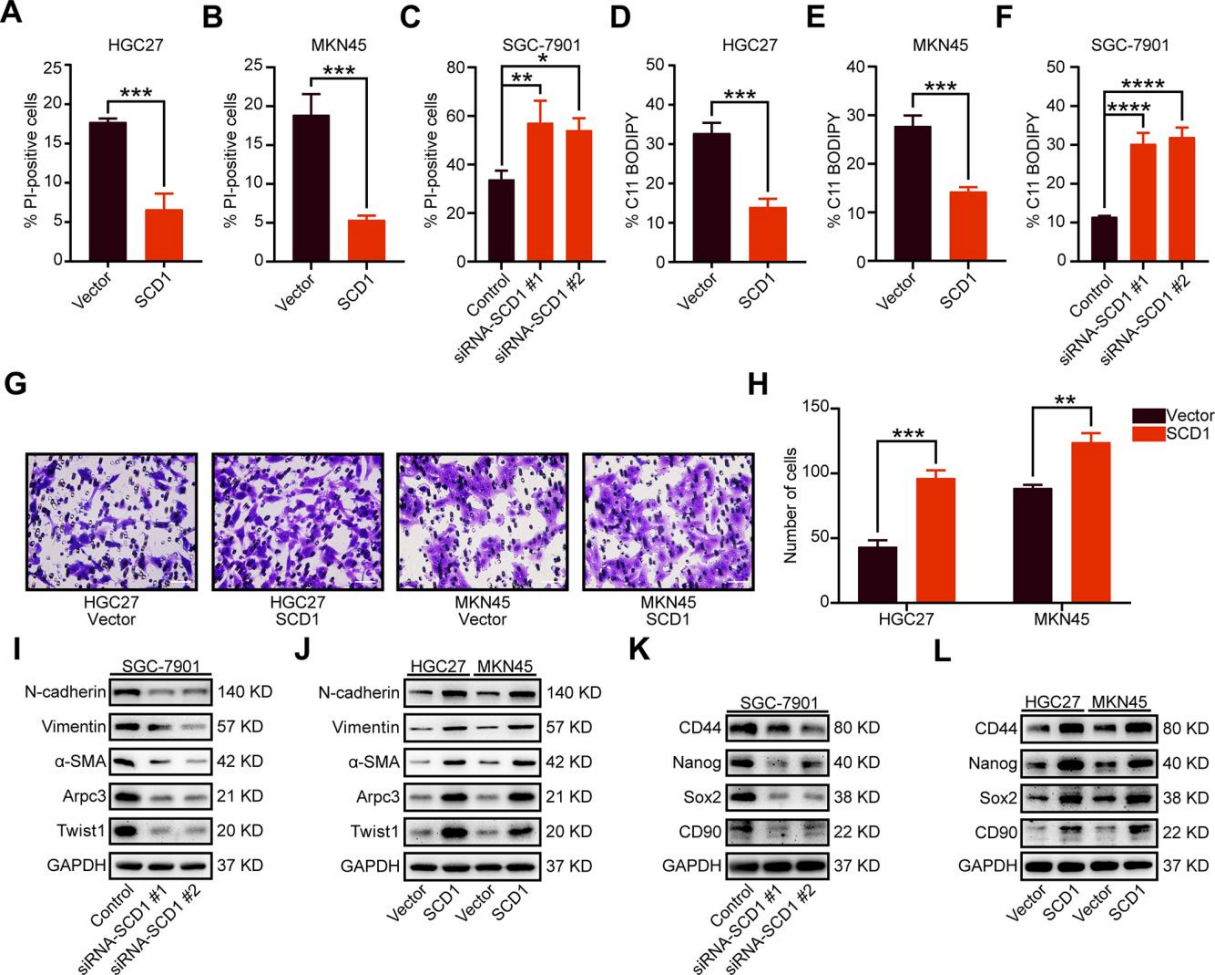
**Oncogenic function of SCD1 in gastric cancer cells.** (**A**–**C**) Gastric cancer cells were treated with 1 μM Erastin for 24 hours, then cell death was assessed using propidium iodide (PI), the bar plot represent quantification of PI-positive cells. Each experiment was conducted in triplicates. (**D**–**F**) C11-BODIPY staining of gastric cancer cells following treatment with 1 μM Erastin for 24 hours, the bar plot represent quantification of C11-BODIPY-positive cells. Each experiment was conducted in triplicates. (**G**, **H**) SCD1 ameliorated the migration ability of gastric cancer cells. (**I**, **J**) The metastatic related markers were analyzed by using western blotting with the indicated antibodies. (**K**, **L**) the cancer stemness related markers were analyzed by using western blotting with the indicated antibodies. GAPDH was used as the internal protein loading control. Each experiment was conducted in triplicates. *, *P* <0.05; **, *P* <0.01; ***, *P* <0.001; ****, *P* <0.0001, respectively.

To identify signaling pathways and biological functions uniquely activated by SCD1 gene, GO, KEGG pathway gene sets and SCD1 expression were analyzed with Gene Set Enrichment Analysis (GSEA) software. Notably, the mean value of SCD1 mRNA expression was considered as the optimal cutoff point. GSEA results showed that KEGG_CELL_CYCLE, KEGG_DNA_REPLICATION, GO_CELL_CYCLE_G1_S_PHASE_TRANSITION AND GO_CYCLIN_DEPENDENT_PROTEIN_SERINE_THREONINE_KINASE_REGULATOR_ACTIVITY were enriched in SCD1 high–expressed group (*P*=0.002, *P*<0.0001, *P*<0.0001 and *P*=0.008; Figure 3B). To validate the function of SCD1 in items mentioned above, SCD1 over-expressed gastric cancer cell models (MKN45-SCD1 and HGC27-SCD1) as well as SCD1 down-regulated gastric cancer cell models (SGC-7901-siRNA-SCD1 #1 and #2) were constructed. As shown in Figure 3C, SCD1 elevated the expression level of cyclin D1 and cyclin E1, which were the markers of “G1-S phase transition”. Additionally, the expression of proliferation-related marker (PCNA), anti-apoptosis marker (Survivin) and anti-ferroptosis markers (SLC7A11 and GPX4) were also enhanced by SCD1. The Wnt-β-catenin signaling pathway and Hippo pathway have been confirmed to be regulated by SCD1 in multiple cancer types [11]., the markers of these two signaling pathways had been validated in gastric cancer cells in this study. As expected, SCD1 could up-regulate the expression of *Yap* and β-catenin as well as the phosphorylation level of β-catenin (Ser-552). *In vitro* cell growth assay also confirmed that SCD1 could enhance the cell proliferation and colony-formation ability of gastric cancer cells (proliferation: HGC27: day5 *P*<0.0001, MKN45 day5 *P*<0.01; Colony formation: HGC27 vector: 152.7 ± 5.364, HGC27 SCD1: 214.0 ± 4.041 *P*<0.001, MKN45 vector: 171.0 ± 3.786, MKN45 SCD1: 352.7 ± 7.055 P<0.0001; Figure 3D–3I). Treated with 1 μM Erastin for 24 hours, the cell death level and lipid oxidation of HGC27, MKN45 and SGC-7901 cells as characteristic features of ferroptosis, were examined and results showed that SCD1 prevented ferroptotic cell death in gastric cancer cells (cell death level: HGC27 vector: 17.72 ± 0.265%, HGC27 SCD1: 6.63 ± 1.153% P<0.001, MKN45 vector: 18.90 ± 1.535%, MKN45 SCD1: 5.36 ± 0.328% *P*<0.001, and SGC-7901 Control: 33.83 ± 2.114%, SGC-7901 siRNA SCD1 #1: 57.17 ± 5.239% *P*<0.01, SGC-7901 siRNA SCD1 #2: 54.13 ± 2.842% *P*<0.05; lipid oxidation: HGC27 vector: 32.80 ± 1.514%, HGC27 SCD1: 13.97 ± 1.235% *P*<0.001, MKN45 vector: 27.80 ± 1.249%, MKN45 SCD1: 14.30 ± 0.529% *P*<0.001, and SGC-7901 Control: 11.47 ± 0.145%, SGC-7901 siRNA SCD1 #1: 30.23 ± 1.637% P<0.0001, SGC-7901 siRNA SCD1 #2: 32.00 ± 1.422% *P*<0.0001 Figure 4A–4F).

### SCD1 enhanced the migration and stemness of gastric cancer cells

Previously studies have shown that SCD1 could accelerate metastasis of colorectal cancer cells [12]. In this study, quantification results revealed that exogenous expression of SCD1 significantly enhanced the migratory activity of gastric cancer cells (HGC27 vector: 43.33 ± 2.848, HGC27 SCD1: 96.33 ± 3.480 *P* <0.001 and MKN45 vector: 88.67 ± 1.453, MKN45 SCD1: 124.00 ± 4.041 *P* <0.01; Figure 4G, 4H). The epithelial-mesenchymal transition (EMT) markers (N-cadherin, Vimentin, α-SMA and Twist1) and cell motility marker (Arpc3) were elevated by high expression of SCD1. Moreover, the expression level of these markers was reduced by silencing of SCD1 (Figure 4I, 4J). Cancer stemness, in our previous study, have been confirmed clearly to be functionally associated with metastasis of gastric cancer [13]. Results of immunoblots assay had shown that SCD1 elevated the expression of cancer stemness related markers (CD44, Nanog, Sox2 and CD90) (Figure 4L), while the reverse tendency had been observed in SCD1 silencing groups (Figure 4K), elucidating the mechanism by which SCD1 ameliorate the metastasis of gastric cancer more intuitively.

### SCD1 accelerated the tumor growth in xenograft mice model

**Figure 5:**
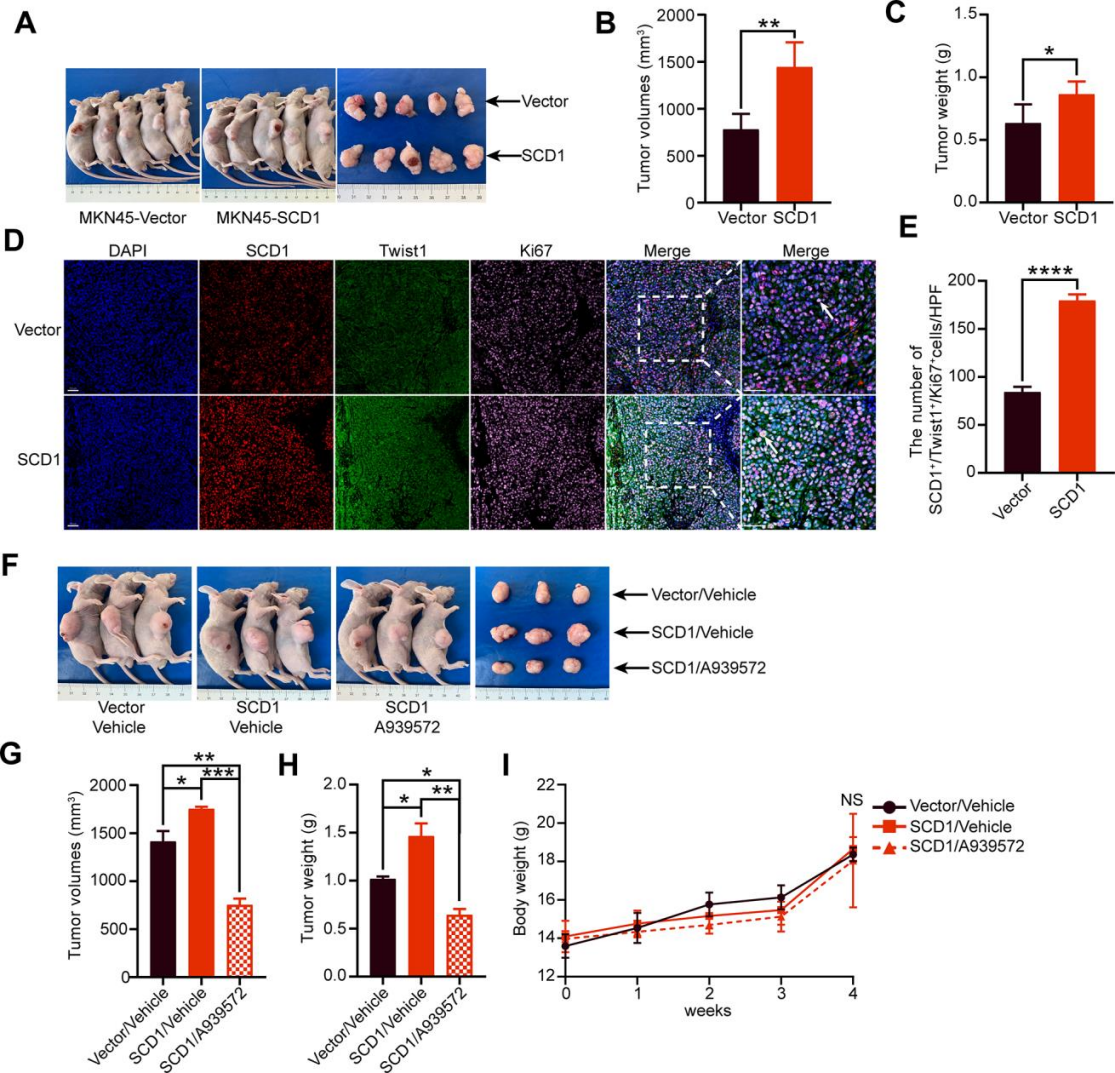
**Oncogenic activity of SCD1 in xenograft mice model.** (**A**) Representative mice and tumor nodules in each group were shown. (**B**) Tumor volumes were analyzed (*n* = 5), results were shown as mean ± SEM (Student *t* test). (**C**) Tumor weights were calculated (*n* = 5), results were shown as mean ± SEM (Student *t* test). (**D**) Immunofluorescent staining of the indicated markers were performed. Scale bar, 50 μm. (**E**) the SCD1, Twist1 and Ki67 positive cells in the tumors were analyzed by using image J software, and results were shown as mean ± SEM (Student *t* test). (**F**) Representative mice and tumor nodules in each group were shown. (**G**) tumor volumes were analyzed (*n* = 3), results were shown as mean ± SEM (the analysis of variance test). (**H**) Tumor weights were calculated (*n* = 3), and results were shown as mean ± SEM (the analysis of variance test). (**I**) Mice were treated by SCD1 inhibitor A939572 or Vehicle i.p. All regimens were administered for twice a week. Body weight was measured weekly during the treatment. There was no obvious decrease in body weight when administration of A939572. *, *P* <0.05; **, *P* <0.01; ***, *P* <0.001; ****, *P* <0.0001; NS, no significance, respectively.

To investigate the effect of SCD1 on the tumorigenesis of gastric cancer cells *in vivo*, the xenograft mice model was constructed and relevant results revealed that SCD1 promoted tumor formation (MKN45-Vector: 779.9 ± 73.670 mm^3^, MKN45-SCD1: 1444 ± 117.300 mm^3^; *P* <0.01, Figure 5A, 5B). Similar to the tendency in tumor volume, the tumor weight of xenografts derived from the MKN45-SCD1 groups were significantly heavier than those of xenografts originating from littermate controls (MKN45-Vector: 0.634 ± 0.067 g, MKN45-SCD1: 0.864 ± 0.045 g; *P* <0.05, Figure 5C). To assess SCD1 activation in gastric cancer at a larger scale, we performed immunofluorescent staining for SCD1, Twist1 and Ki67 of paraffin-embedded tissue sections from MKN45-SCD1 xenografts and control ones. Interestingly, tumor tissues from MKN45-SCD1 mice showed a significantly increased number of Twist1^+^ and Ki67^+^ cells, indicating SCD1 enhanced proliferation and metastasis as compared with tumor tissues from control ones (MKN45-Vector: 84.200 ± 2.478, MKN45-SCD1: 179.400 ± 2.926, *P* <0.0001; Figure 5D, 5E).

To test whether blocking of SCD1 could alleviate tumorigenesis of gastric cancer cells *in vivo*, we induced gastric cancer cells in mice by injecting MKN45-SCD1 cells subcutaneously as described above. After one week, mice were treated with either vehicle or the SCD1 inhibitor A939572, and sacrificed after 3 weeks. Both tumor weight and volume were assessed. As shown in Figure 5F, 5G, inhibition of SCD1 exerted substantial antitumor effects, and reduced both tumor volume and tumor weight compared with MKN45 Vector/vehicle group and MKN45 SCD1/vehicle group (tumor volume: MKN45 Vector/vehicle 1413 ± 107.8 mm^3^, MKN45 SCD1/vehicle 1752 ± 22.13 mm^3^, MKN45 SCD1/A939572 751.5 ± 38.78 mm^3^; tumor weight: MKN45 Vector/vehicle 1.017 ± 0.027 g, MKN45 SCD1/vehicle 1.463 ± 0.132 g, MKN45 SCD1/A939572 0.643 ± 0.060 g; Figure 5F–5H). Moreover, no signs of toxicity as evidenced by body weight measurement were observed (4 weeks: MKN45 Vector/vehicle 18.36 ± 0.358 g, MKN45 SCD1/vehicle 18.66 ± 0.358 g, MKN45 SCD1/A939572 18.05 ± 1.407 g, MKN45 SCD1/vehicle vs. MKN45 SCD1/A939572 P >0.05, MKN45 Vector/vehicle vs. MKN45 SCD1/A939572 P >0.05 Figure 5I).

Collectively, this study demonstrated that SCD1 could promote tumor growth of gastric cancer cells and protect them from ferroptotic cell death. Furthermore, gastric cancer patients with high-expressed SCD1 might have less-optimistic prognosis.

## DISCUSSION

Here we described the expression and pro-tumorigenic function of SCD1 in gastric cancer. By using *in vivo*, *in vitro*, and *in silico* methods, our results revealed that SCD1 was an oncogene in gastric cancer, and exhibited the ability to ameliorate metastasis, anti-ferroptotic cell death and growth of gastric cancer cells *in vitro* and *in vivo*.

Gastric cancer is the third most common cancer worldwide and obsessed by less-optimistic prognosis [14–16]. One risk factor of gastric cancer is metabolic disorder, such as cholesterol syndrome [17, 18]. The alterations of lipid uptake and metabolism during tumorigenesis, have been reported to be linked to the survival and metastatic potential of cancer cells. Unlike in cancer cells with *de novo* lipogenesis in itself, normal cells mainly absorb circulating lipids [6]. Dividing cells need plenty of fatty acids to maintain the fluidity of cell membrane as structural components, to serve as energy stores and signaling lipids. Therefore, proliferating cancer cells could be identified by a greater demand for MUFAs. Meanwhile, an increase in the enrichment of MUFAs as well as the simultaneous reduction of SFAs and PUFAs have been observed in tumor tissues of various origins (ovarian, lung, liver cancer) [5, 19, 20]. During the *de novo* lipogenesis, cytoplasmic acetyl-CoA were carboxylated and then catalyzed by fatty acid synthase (FASN), which finally produces the saturated FA palmitate (FA 16:0). Then, SCD1 desaturated palmitate into monounsaturated FAs (MUFAs) [21].

Metabolic layer is at the interface between other cellular processes in the cells [1]. During the initiation of primary tumor, the formation of lipid rafts which increase membrane cholesterol concentrations are necessary for activation of pro-proliferative and growth-stimulatory signaling pathways, such as accelerating the accumulation of HER2, IGF-1 to induce PI3K-Akt signaling pathway [22]. While in the process of metastasis, cancer cells efflux cholesterol to keep itself at low membrane concentrations, in order to promote plasma membrane fluidity and epithelial-mesenchymal transition (EMT) [21]. Moreover, fatty acids could also serve as secondary messengers in signal transduction pathways to maintain cellular proliferation and survival. For instance, triphosphorylated PI(3-5)P3 (PIP_3_) could facilitate the transition of Akt to plasma membrane, and lead to subsequent activation including phosphorylating and inhibiting many pro-apoptotic proteins (BAD, procaspase-9 and FOXO transcription factors), which could modulate the expression of apoptotic enzymes [23]. Furthermore, numerous studies have closely linked the lipid metabolism with cancer stem cell [24]. Concretely, fatty acid oxidation (FAO) is activated by stem cell factor Nanog, which promotes the stem-like property of hepatocellular carcinoma (HCC) [25], and elevates levels of lipid droplets (LDs) as a distinctive feature of CD133^+^ colorectal CSCs [24]. As for SCD1, it is necessary for the tumor sphere formation and expression of stem cell markers including ALDH1A1, Oct4 and Nanog. Mechanistically, SCD1-mediated regulation of cancer stemness is linked to the Hippo signaling pathway and Wnt-β-catenin pathway [24]. Inhibition of SCD1 by small molecular inhibitors or shRNA could significantly promote ferroptosis and apoptosis or inhibit the growth, migration and invasion of cancer cells. Additionally, MUFAs including oleic acid could reverse the effect of decreased SCD1 expression in ovarian, clear cell renal cell carcinoma and colorectal cancer [5, 8, 12]. Besides, a combined pharmacological approach that targeting SCD1 could counteract the chemo-resistance of cancer cells and elevate the therapeutic efficacy of commonly used chemotherapeutic and targeted drugs, such as gefitinib, sorafenib and cisplatin [7, 26, 27].

Several shortcomings should be mentioned as follow. First and foremost, the study was retrospective in nature and all of the clinicpathological data were accessed from TCGA-STAD database. Thus, external cohorts were needed to validate the predictive accuracy of the nomogram for OS. Secondly, as SCD1 inhibitor MK-8245 for the treatment of type II diabetes had been done in the clinical trial (NCT00790556) and relevant results revealed that no serious adverse events occurred in all patients evaluated [28]. Therefore, more prospective study should be conducted in further investigation about the treatment value of SCD1 inhibitor in cancer patients, especially in gastric cancer patients.

## CONCLUSIONS

In conclusion, our study demonstrated that SCD1 could accelerate the migration, anti-ferroptotic cell death and growth of gastric cancer cells, and predict less-optimistic prognosis in gastric cancer patients. Our work illustrates the potential of SCD1 as biomarker in early diagnosis as well as a therapeutic targets of gastric cancer.

## MATERIALS AND METHODS

### *In vitro* gastric cancer cell line culture

Human gastric cancer cell lines (SGC-7901, MKN45 and HGC27) were used for all experiments. All cells were purchased from the Chinese Academy of Science, authenticated routinely by short tandem repeat (STR) profiling and tested to exclude mycoplasma contamination prior to use. All gastric cancer cells were passaged for only a maximum of three months after resuscitation, and cultured in Dulbecco’s Modified Eagle Medium (DMEM) (BasalMedia, cat: E210702) supplemented with 10% fetal bovine serum (FBS), 100 U/ml Penicillin G and 100 μg/ml streptomycin sulfate, and maintained at a 37°C incubator with 5% CO_2_ atmosphere.

### SCD1 silencing and virus transduction

For RNA interference, SGC-7901 cells were transfected with small interfering RNAs (siRNAs) against human SCD1 with Lipofectamine 2000 (Invitrogen, cat: 11668-019) according to the manufacturer’s instructions. The siRNA sequences for SCD1 were designed and synthesized by GenePharma, and the sequences were listed as following: #1: forward: 5’-CACAUGCUGAUCCUCAUAATT-3’, reverse: 5’-UUAUGAGGAUCAGCAUGUGTT-3’; #2: forward: 5’-GGUACUACAAACCUGGCUUTT-3’, reverse: 5’-AAGCCAGGUUUGUAGUACCTT-3’.

### SCD1 silencing was confirmed by western blotting

For virus transduction, cells were transducted with polybrene, according to the manufacturer’s instructions and selected with 5 μg/ml puromycin (Invivogen, cat: Ant-pr-5). The SCD1 gene was constructed in lentiviral vector (pWPXL). Overexpression of SCD1 was confirmed by western blot. The stable cell lines which SCD1 had been overexpressed were then used for further experiments.

### Western blotting

Western blotting was performed as previously described [13]. In brief, cells were lysed in standard Radioimmune-Precipitation Assay (RIPA) buffer containing 1% protease inhibitor cocktail (Sigma-Aldrich, cat: P8340) and 10 μM sodium fluoride. The protein samples were separated using sodium dodecyl sulfate polyacrylamide gel electrophoresis (SDS-PAGE, 12.5%) and transferred onto polyvinylidene fluoride (PVDF) membranes. Then, the membranes were blocked with 5% BSA in TBS-T, and incubated for corresponding antibodies overnight at 4°C, followed by the corresponding secondary antibodies. The immunoreactive proteins on the membranes were then visualized using the infrared imaging system (LI-COR Biosciences) and ECL substrate solution (NCM Biotech, cat: P10300). The band intensity was densitometrically evaluated by Image J software (NIH). The antibodies were listed in Supplementary Table 1.

### Purification of endoplasmic reticulum

Endoplasmic reticulum (ER) fraction was isolated using the ER isolation kit (Sigma-Aldrich; cat: ER0100), according to the manufacturer’s protocol. Purity of cell fractions was examined by western blotting against SERCA2 (ER marker).

### Flow cytometric detection of ROS

Cells were seeded in 6-well plates overnight and subjected to Erastin (1 μM) for 24 hours. Cells were then incubated with 5 μM C11-BODIPY (Thermo Fisher Scientific, cat: D-3861) for 30 min. After that, cells were washed with PBS, trypsinized and neutralized with 10% FBS in PBS at 1:1 volume. For flow cytometry, signal was analyzed in the FITC channel and Software analysis was carried out using FlowJo v10.

### Determine of cell death

Cells were seeded in 6-well plates overnight and subjected to Erastin (1 μM) for 24 hours. Then cells were harvested and stained with 2 μg/ml propidium iodide (PI). Dead cells (PI-positive cells) were analyzed using flow cytometer and software analysis was carried out using FlowJo v10.

### Transwell assay

Assays were performed as described previously [13], In brief, Fifty thousand cells were seeded in the upper compartment of the Transwell chambers with membranes containing 8-μm pores (Millipore. cat: MCEP24H48). The cells on the upper side of the membrane were removed and washed in PBS buffer after 24 h. Cells migrating to bottom side of the membrane were fixed and stained using 0.1% crystal violet (Sigma-Aldrich). Pictures of five random fields at a 200× magnification were captured, and the experiments were performed in triplicates.

### *In vitro* cell growth assays

Both assays were performed as described previously [29]. 1000 cells were plated into the indicated well of 96-well plates (Eppendorf, cat: 0030730119) and cultured overnight. CCK-8 (Dojindo Laboratories, cat: LQ730) was added into the indicated wells and then incubate at 37°C for 2 h. The absorbance at 450 nm was detected by microplate spectrophotometer (BioTek) to count the amount of vital cells in certain wells, for which the process will last for five days. In order to determine the effects over an extended period of time, one thousand cells were seeded into a 6-well plate in complete medium and incubated at 37°C for 14 days followed by fixed in 70% ethanol and stained with 0.1% crystal violet. All experiments were performed in triplicates.

### Bioinformatic analyses

### Data acquisition

The mRNA expression data and clinical information of gastric cancer patients were downloaded from The Cancer Genome Atlas (TCGA) in January 2016. The samples which contained “0” gene expression values, incomplete survival information or pathological characteristics were excluded. Above processes were executed in R, using “RTCGA Toolbox” R packages. Conventional clinic pathological factors containing age, gender, gastric cancer stage (AJCC), lymph node metastasis and T phase were recorded and described in Supplementary Table 2. Furthermore, the differential mRNA expression level of SCD1 between a variety of cancer tissues and normal ones were obtained from the TCGA and GEO database (GSE13911 and GSE19826) and analyzed by the UALCAN web tool (http://ualcan.path.uab.edu/) [30].

### The Human Protein Atlas (HPA)

The immunohistochemistry images of SCD1 in gastric cancer tissues of the patient (ID: 2066) was downloaded from the Human Protein Atlas (HPA) (http://www.proteinatlas.org/) [31].

### The Kaplan-Meier plotter

The prognostic value of SCD1 in gastric cancer patients was analyzed using the GEO database [32], which contained GSE14210, GSE15459, GSE22377, GSE29272 and GSE51105. And the relationship between SCD1 expression and the prognosis of sarcoma, bladder carcinoma, kidney renal papillary cell carcinoma and cervical squamous cell carcinoma patients were analyzed using the TCGA database [33]. The hazard ratio with 95% confidence intervals and log rank *p*-value were calculated as well.

### Identifying the correlated factors interacted with SCD1

The cBioportal, Gepia and MEM analysis tools were applied to identify the factors correlated with SCD1 [34, 35], the common factors existed in above three analysis tools were shown in venn diagram (http://bioinformatics.psb.ugent.be/webtools/Venn/). The expression relationship between SCD1 and correlated factors were evaluated using spearman correlation analysis, and then the above mentioned factors were uploaded into Database for Annotation, Visualization, and Integrated Discovery (DAVID) web tool (https://david.ncifcrf.gov/home.jsp) [36]. Both Kyoto Encyclopedia of Genes and Genomes (KEGG) pathway enrichment and Go function enrichment analysis were utilized. *P* value <0.05 was considered as the cutoff criteria.

### Analysis of gastric cancer data in cBioportal for Cancer Genomics database

SCD1 were analyzed by cBioportal data, which is an open-access downloaded bio-database. The primary search parameters included alterations (Mutation, amplification, and deep deletion) and predicted mutation site with the default setting across samples curated from stomach adenocarcinoma.

### Gene set enrichment analysis (GSEA)

GSEA (http://software.broadinstitute.org/gsea/index.jsp) was applied to find biological function of SCD1 genes. Annotated gene sets c2.cp.kegg.v5.2.symbols.gmt, and GO gene sets were chosen as the reference gene sets. The expression level of SCD1 were selected as a phenotype label. The false defect rate q value < 0.05 and normalized enrichment score (NSE) > 1were identified to sort the pathways and items enriched in each phenotype [37].

### Xenograft experiments and immunofluorescent staining of tissue sections

Male BALB/c mice (4-weeks-old) were purchased from Institute of Zoology Chinese Academy of Sciences (Shanghai, China), and housed at a specific pathogen-free environment. Then, mice were randomly divided into 2 groups (5 mice in each group) in the first experiment, while divided into 3 groups (3 mice in each group) in the second experiment. Our experiments strictly abided to the ethical guidelines of the Ethics Committee of Research Center of Experimental Medicine, Shanghai Jiaotong University School of Medicine Affiliated Ruijin Hospital. The MKN45-SCD1 cells and their negative control ones were trypsinized and re-suspended in 100 μl PBS which contained 1.5 million gastric cancer cells and inoculated subcutaneously. The next week, mice in the treatment group were injected with 20 mg/kg A939572 i.p. Mice in the Vector and SCD1 groups were injected with 2% DMSO i.p. Treatment was administered for twice a week and last for 3 weeks.

Tumor nodules were measured weekly after the length exceeded 2 mm, and the tumor volume was calculated from the formula V = (Width^2^ × Length)/2 (V = volume). Mice were sacrificed by CO_2_ asphyxiation and cervical dislocation at 4 weeks, and xenografts were measured by immunofluorescence. Carl Zeiss microscope (ZEISS Company) was used to capture images of the periphery of the tumor. The area staining for SCD1, Twist1 and Ki67 were then defined as a percentage of the area staining for DAPI using Image J software (NIH).

### Statistical analysis

In this study, 5.43 was defined as the cutoff value of SCD1 for high and low expression according to the analysis of X-tile software [38] based on its relationship with overall survival. Univariate Cox regression analysis was utilized to identify independent prognostic variables based on SCD1 level and other clinical characteristics, including age, gender, AJCC stage, T classification and lymph node metastasis. *P* < 0.05 was set as the cutoff *p* value to select the factors from the univariate analysis data to execute the multivariate model, and a forward stepwise Cox regression model was utilized to select independent prognostic factors. The nomogram was applied using the data of SCD1, Age, AJCC stage and the package of rms in R version. The predictive accuracy of the nomogram was checked by concordance index (C-index) and assessed by calibration comparing nomogram-predicted with observed Kaplan-Meier estimates of survival probability. The C-index was positively related with the prognostic prediction. Time-dependent AUC analysis represented an extension of the ROC curve that assess the discriminatory power of a prognostic model for time-dependent cancer outcomes. In order to compare the ROC curves visually, the area under the ROC curve (AUC) was analyzed. For each time point, the AUC value estimated the probability that a dead cancer patient was classified into a higher staging category than the one alive. Variance between groups were compared by Mann-Whitney *u*-test, one-way analysis of variance (ANOVA) or unpaired Student’s *t*-test. Data were shown as mean and standard error of the mean (SEM). All statistical analysis were performed by statistical programming language R for windows (cran.r-project.org). Two-tailed *P*-value less than 0.05 were considered as statistically significance.

## Supplementary Material

Supplementary Tables
