# Supplementary material for: Stearoyl-CoA desaturase 1 (SCD1) facilitates the growth and anti-ferroptosis of gastric cancer cells and predicts poor prognosis of gastric cancer
Source: Aging (Albany NY). 2020 Jul 29;12(15):15374–91. doi: 10.18632/aging.103598 (PMC7467382; doi:10.18632/aging.103598)
Supplement: Supplementary Tables [file aging-12-103598-s001..pdf]

SUPPLEMENTARY TABLES

Supplementary Table 1. Antibodies, source and dilution.

Antibody	Source	Dilution (application)
CD44	Cell Signaling Technology; #3570	1:1000(WB)
Survivin	Cell Signaling Technology; #2808	1:1000(WB)
TWIST1	Abclonal; #A3237	1:1000(WB) 1:100(IF)
N-cadherin	Cell Signaling Technology; #14215S	1:1000(WB)
Nanog	Abclonal; #A14150	1:1000(WB)
Sox2	Abclonal; #A11501	1:1000(WB)
Arpc3	Abclonal; #A7767	1:1000(WB)
α-SMA	Abcam; #AB5694	1:1000(WB)
CD90	Santa Cruz; #SC-19614	1:1000(WB)
GAPDH-Mouse	Cell Signaling Technology; #51332	1:1000(WB)
DAPI	Beyotime; #C1002	1:1000(IF)
GPX4	Abclonal; #A13309	1:1000(WB)
SLC7A11	Proteintech; #26864-1-AP	1:1000(WB)
β-catenin	Cell Signaling Technology; #8480S	1:1000(WB)
Phospho-β-catenin-Ser552	Abclonal; #AP0579	1:1000(WB)
SCD1	Abclonal; #A16429	1:1000(WB)
Cyclin D1	Cell Signaling Technology; #55506S	1:1000(WB)
Cyclin E1	Cell Signaling Technology; #4129S	1:1000(WB)
PCNA	Abclonal; #A0264	1:1000(WB)
Vimentin	Cell Signaling Technology; #5741S	1:1000(WB)
YAP	Cell Signaling Technology; #14074	1:1000(WB)
SERCA2	Proteintech; #67248-1-lg	1:1000(WB)

Supplementary Table 2. Clinicopathological characteristics of patient samples and expression of SCD1 in TCGA-STAD database.

Characteristics	Number of cases (%)
Gender	
Male	218(63.4)
Female	126(36.6)
Age(year)	
≤65	152(44.2)
>65	192(55.8)
T phase	
I	16(4.7)
II	70(20.3)
III	161(46.8)
IV	97(28.2)
Lymph node metastasis	
0	107(31.1)
I	88(25.6)
II	72(20.9)
III	77(22.4)
AJCC Stage	
I	49(14.2)
II	110(32)
III	148(43)
IV	37(10.8)
SCD1	
Low	68(19.8)
High	276(80.2)
